# Neutrophil elastase as a biomarker for bacterial infection in COPD

**DOI:** 10.1186/s12931-019-1145-4

**Published:** 2019-07-30

**Authors:** Samantha J. Thulborn, Vijay Mistry, Christopher E. Brightling, Kelly L. Moffitt, David Ribeiro, Mona Bafadhel

**Affiliations:** 1 Respiratory Medicine Unit, Nuffield Department of Medicine, John Radcliffe Hospital, University of Oxford, Level 7, Oxford, OX3 7DU UK; 2 Oxford NIHR Biomedical Research Centre, University of Oxford, Oxford, UK; 3 Institute of Lung Health, University of Leicester, Leicester, UK; 4 ProAxsis Ltd, Belfast, UK

## Abstract

**Background:**

Chronic obstructive pulmonary disease (COPD) is predominantly associated with neutrophilic inflammation. Active neutrophil elastase (NE) is a serine proteinase, secreted by neutrophils, in response to inflammation and pathogen invasion. We sought to investigate if NE could be used as a biomarker for bacterial infection in patients with COPD.

**Methods:**

NE was quantified using ProteaseTag® Active NE Immunoassay (ProAxsis, Belfast) from the sputum of COPD subjects at stable state, exacerbation and 2 weeks post treatment visit.

**Results:**

NE was measured in 90 samples from 30 COPD subjects (18 males) with a mean (range) age of 65 (45–81) years and mean (SD) FEV_1_ of 47% (18). The geometric mean (95%CI) of NE at stable state was 2454 ng/mL (1460 to 4125 ng/mL). There was a significant increase in NE levels at an exacerbation (*p* = 0.003), and NE levels were higher in a bacterial-associated exacerbation (NE log difference 3.873, 95% CI of log difference 1.396 to 10.740, *p* = 0.011). NE was an accurate predictor of a bacteria-associated exacerbation (area (95%CI) under the receiver operator characteristic curve 0.812 (0.657 to 0.968).

**Conclusion:**

NE is elevated during exacerbations of COPD. NE may be a viable biomarker for distinguishing a bacterial exacerbation in patients with COPD.

**Trial registration:**

Leicestershire, Northamptonshire and Rutland ethics committee (reference number: 07/H0406/157).

## Background

Chronic bacterial infection may play an important role in the progression of COPD [1]. Bacterial products cause epithelial injury and remodeling, stimulate an inflammatory response and disrupt elastolytic activity, leading to degradation of tissue within the airways [2]. The inflammatory response to a bacterial infection is predominantly driven by neutrophils [3, 4]. Neutrophils have many roles within the immune system one of which is in the defense against pathogens [5]. However neutrophils are currently not quick or easy to measure [6] and due to their multiple roles within the immune system are not a viable biomarker for a bacterial exacerbations in COPD [7].

Neutrophil elastase (NE) is a serine protease [8] stored and secreted by neutrophils [9]. Serine proteases aid in the killing of pathogens, both intracellularly and via extracellular traps [10, 11] and are only activated upon appropriate signaling [12]. NE has been shown to have an essential role in the immune defense against microbial infection [13] and has been shown to be a key mediator in tissue remodeling and inflammation [14, 15]. In patients with Bronchiectasis and Cystic Fibrous, NE has been associated with exacerbations, lung function decline and disease severity [16] [17]. A study focusing on NE in Bronchiectasis found that NE levels were elevated at an exacerbation state and responded to antibiotics [16]. The inhibition of NE improves lung function and airway inflammation [18], NE inhibitors and their therapeutic applications are being considered [19].

We now recognise that COPD exacerbations are heterogeneous events [3, 20] and that bacteria play a role in up to 50% of moderate non-severe exacerbations [20]. In patients with COPD, antibiotics are recommended as treatment of an infective exacerbation however antibiotic resistance is a growing concern [21, 22]. NE activity in COPD and during exacerbations is understudied. We investigated if NE in patients with COPD is associated with clinical outcomes and is a viable biomarker for bacteria-associated exacerbations.

## Materials and methods

### Subjects and sampling

A sufficient sputum sample for active NE assay was available from 30 COPD subjects at stable state, exacerbation and 2 weeks post treatment from a previously published study; subject inclusion and exclusion criterion, study design and measurements have been previously described [20]. In brief, subjects were sampled at stable state at the onset of an exacerbation and 2 weeks post treatment. Stable state was defined as one where subjects were free from an exacerbation for at least 6 weeks prior to the visit. At each visit, pre and post bronchodilator spirometry, health status questionnaires, blood and sputum samples were obtained. Health status and symptoms were measured using the chronic respiratory disease questionnaire (CRQ) [23] and the visual analogue score (VAS) [24]. An exacerbation event was defined according to Anthonisen criteria [25] with all measurements conducted prior to treatment. Exacerbations were treated according to NICE guidelines [26] with either antibiotics, corticosteroids, or both. All subjects gave written informed consent and the study was approved by the Leicestershire, Northamptonshire and Rutland ethics committee (reference number: 07/H0406/157).

### Sputum processing

Sputum processing was carried out upon collection at the University of Leicester. This involved plug selection, followed by a dispersion step with Dulbecco phosphate-buffered saline (PBS) and a mucolytic step with dithiothreitol (DTT); sputum supernatants were stored at each step at − 80 °C. A filtration step to remove debris for cytospin preparation and quantification of cell differential count was then performed. Samples with cell viability of < 40% and squamous contamination of > 20% were excluded. A proportion of filtrate (100 μL) was removed for the quantification of colony forming units (CFU) to measure bacterial load. The sample was serially diluted and plated on chocolate and blood agar plates and left to grow for 24 h at 5% CO_2_ and 37 °C. Bacterial colonisation was defined as a bacterial load of CFU > 10^5^ in a sample collected at stable state. An acute bacterial infection was defined as either i) an elevated bacterial load (colony forming unit of > 10^7^); and/or ii) a positive sputum culture at the exacerbation visit.

### Active neutrophil elastase immunoassay

The detection of NE levels was carried out at the University of Oxford utilising the ProteaseTag® Active Neutrophil Elastase Immunoassay kit (ProAxsis, Belfast). Samples were diluted 1 in 10, were run in duplicate and the standard concentrations ran from 15.63 ng/mL to 1000 ng/mL and were fit to a 4-parameter logistic fit for analysis. The lower limit of detection of this assay is 7.2 ng/mL. The effect of DTT on the NE assay, was analysed by measuring paired DTT and PBS supernatant sputum samples (*n* = 5). No difference between NE levels were detected in the DTT and PBS sputum treated samples (*p* = 0.867). All remaining analyses were thus performed on DTT treated sputum.

### Statistical analysis

GraphPad Prism version 6 (GraphPad Software Inc., La Jolla, CA, USA) and SPSS Statistics version 22 (SPSS Inc. Chicago, IL, USA) was used for statistical analysis. The Kolmogorov-Smirnov test was applied to test for normality. Parametric data is presented as mean (SD) or geometric mean (95%CI) for log transformed data unless stated. Non-parametric data is presented as median (IQR). Paired and unpaired t-test and one-way analysis of variance (ANOVA) tests were used to compare variables. Receiver operating characteristic (ROC) curves were calculated to measure the sensitivity and specificity of NE as a biomarker for bacterial colonisation and a bacteria-associated exacerbation. A probability of *p* < 0.05 was considered as the threshold of significance.

## Results

The baseline participant characteristics are presented in Table 1. The geometric mean (95%CI) of NE at stable state was 2454 ng/mL (1460 to 4125 ng/mL). NE at stable state did not relate to use of inhaled corticosteroids (*p* = 0.731), disease severity (*p* = 0.874), determined by the global initiative for chronic obstructive lung disease (GOLD [27]) or exacerbation frequency (*p* = 0.992). There was no significant difference in NE levels when looking at the number of co-morbidities a subject had (*p* = 0.668). There was no correlation of NE with any clinical outcomes measured presented in Table 2. There was a correlation of NE with measures of systemic inflammation (CRP r = 0.373; *p* = 0.042) and airway inflammation (sputum total cell count r = 0.553, *p* = 0.002; and sputum absolute neutrophil count r = 0.515, *p* = 0.004) but not the percentage of sputum neutrophils (r = 0.312, *p* = 0.099). In 5 subjects with available sputum samples longitudinally at stable state, NE levels were found to be repeatable (repeated measures ANOVA *p* = 0.724). NE at stable state was a poor predictor of chronic bacterial infection with an area under the receiver operator curve of 0.555 (95%CI 0.327 to 0.783, *p* = 0.628).

**Table 1:** Clinical characteristics at stable baseline state

Subjects, n	30
Male, *n* (%)	18 (60)
Age (Year) ¥	65 (45–81)
Smokers, n (%)	14 (47)
Pack year history ¥	50 (10–156)
On ICS treatment (%)	20 (90)
Co-morbidity – Cardiovascular (%) *	9 (31)
Co-morbidity – Diabetes (%)*	3 (10)
Co-morbidity – Endocrine (%)*	0 (0)
Co-morbidity – Depression (%)*	6 (21)
Co-morbidity – Osteopenia/porosis (%)*	6 (21)
Co-morbidity – Anaemia (%)*	0 (0)
GOLD 2, n (%)	9 (30)
GOLD 3, n (%)	16 (53)
GOLD 4, n (%)	5 (17)
Post-bronchodilator FEV_1_ (L)	1.25 (0.52)
Post-bronchodilator FEV_1_% predicted	47 (18)
FEV/FVC ratio, %	47 (12)
Chronic respiratory disease questionnaire, units	4.11 (0.99)
Total visual analogue scale, mm	156 (77)
Proportion taking inhaled corticosteroids, n (%)	27 (90)
Sputum total cell count, ×10^6^/g	3.50 (2.11 to 5.82)
% neutrophil sputum count	77 (20)
Total sputum neutrophil count, ×10^6^/g	2.80 (1.58 to 4.96)
Proportion with positive microbiology culture, n (%)	9 (30)
Colony forming units, ×10^7^	1.09 (0.34 to 3.48)

*GOLD* - Global Initiative for chronic obstructive lung disease individuals grouped (1–4) by severity of disease; FEV_1_ – Forced expiratory volume in 1 s; FVC - Forced vital capacity; Chronic Respiratory Disease Questionnaire, scores range between 1 to 7 with higher score representing better health quality; Visual Analogue Scale, performed on 100 mm line from ‘no symptoms’ to ‘worst symptoms’, higher scores represent worse symptoms (total score addition of measured domains: cough, dyspnoea, sputum production and sputum purulence). Unless indicated all tables are mean and standard deviation in brackets. ¶ - Geometric mean and 95% Confidence Intervals. ¥- Mean (range). € Median (IQR). Three patients had no sample available for microbiology culture.* One patient missing co-morbidity data

**Table 2:** Correlations of clinical outcomes with NE levels in sputum

	R value	*P* value
Post-bronchodilator FEV_1_ (L)	0.04	0.81
FEV/FVC ratio, %	−0.02	0.94
Chronic respiratory disease questionnaire, units	0.09	0.63
Total visual analogue scale, mm	0.11	0.58

*FEV*_*1*_ – Forced expiratory volume in 1 s; *FVC* - Forced vital capacity; Chronic Respiratory Disease Questionnaire, scores range between 1 to 7 with higher score representing better health quality; Visual Analogue Scale, performed on 100 mm line from ‘no symptoms’ to ‘worst symptoms’, higher scores represent worse symptoms (total score addition of measured domains: cough, dyspnoea, sputum production and sputum purulence)

### NE levels at exacerbation

**Fig. 1:**
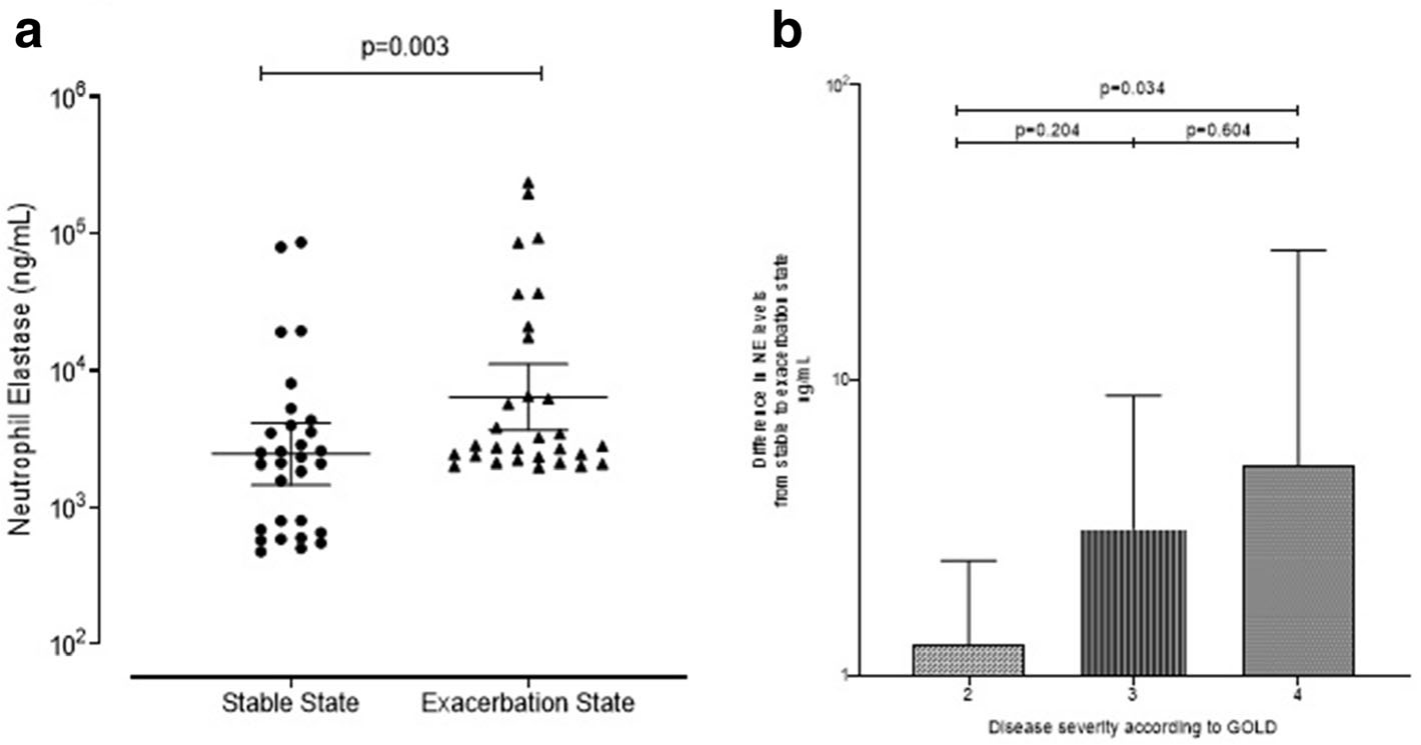
Levels of NE in sputum at stable state and then at an exacerbation in 30 subjects with COPD. Horizontal bars at mean (95% CI) (**a**). The difference in NE levels from stable to exacerbation state according to GOLD. Mean (95% CI) (**b**)

**Fig. 2:**
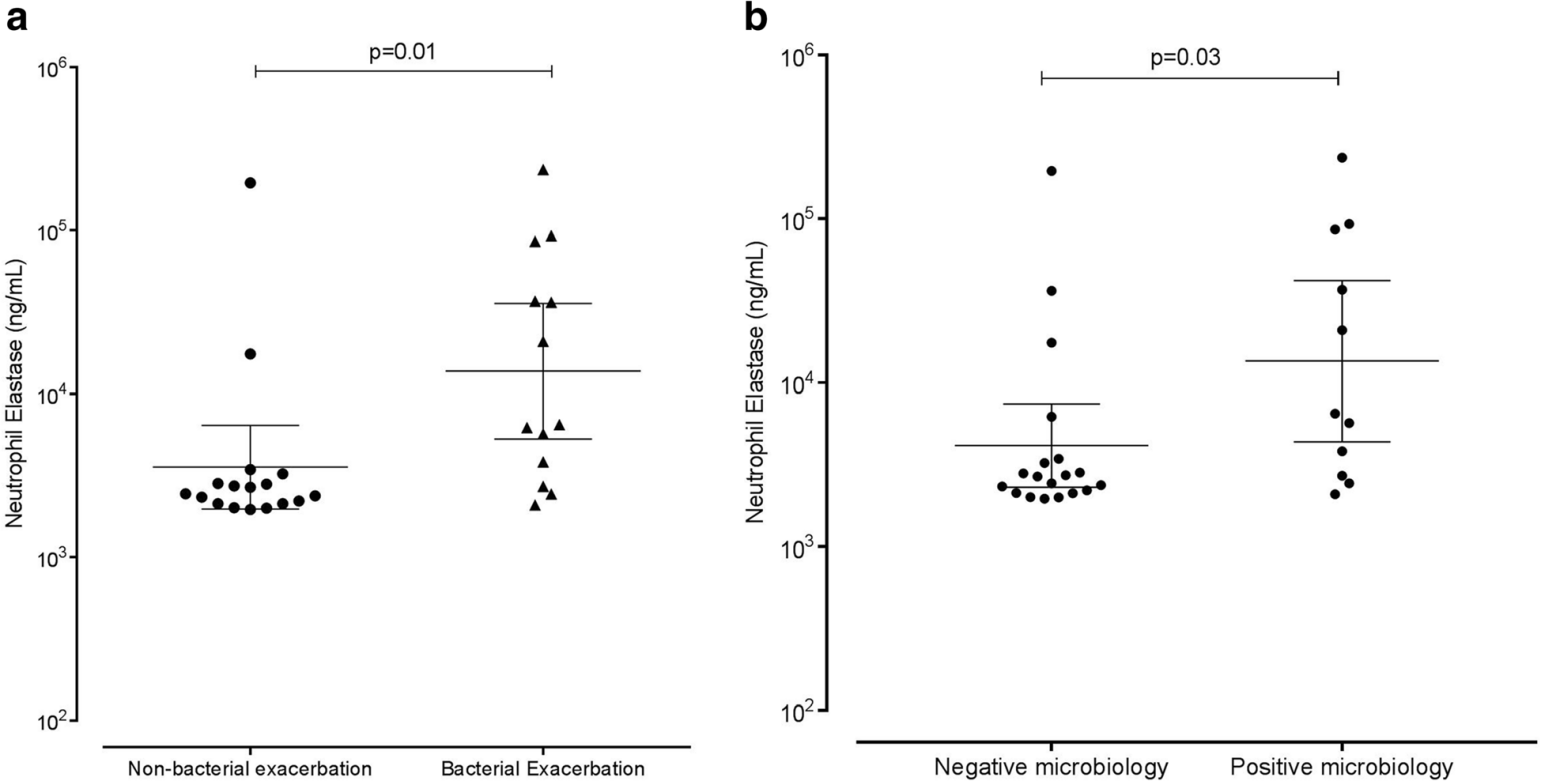
Levels of sputum NE in subjects with a non-bacterial or bacterial exacerbation (**a**) and in positive versus negative culture at exacerbations (**b**). Horizontal bars at mean (95% CI)

The geometric mean (95%CI) of NE at exacerbation state was 6400 ng/mL (3664 to 11,180 ng/mL) and was significantly increased compared to stable state (Log difference 2.606, 95%CI of log difference 1.416 to 4.808 *p* = 0.003) (Fig. 1a). We then separated this in disease severity using GOLD criteria and looked at the change in NE levels from stable to an exacerbation state. A significant difference was seen between NE levels at GOLD 2 compared to GOLD 4 (Log difference 4.046, 95%CI 1.138 to 14.388, *p* = 0.034), with GOLD 4 having a significantly larger increase in NE from stable to exacerbation state (Fig. 1b). At exacerbation, NE correlated with FEV_1_ and FVC (r = − 0.381, *p* = 0.038 and r = − 0.470, *p* = 0.010, respectively) but not symptom scores (CRQ r = 0.027, *p* = 0.385 and VAS r = 0.076, *p* = 0.142) or exacerbation frequency (*p* = 0.495). NE correlated with airway inflammation (sputum total cell count r = 0.414, *p* = 0.023 and sputum absolute neutrophil count r = 0.457, *p* = 0.013) in addition to the percentage of sputum neutrophils (r = 0.496, *p* = 0.006). NE correlated with both CRP and peripheral blood neutrophil counts (r = 0.388, *p* = 0.038 and r = 0.538, *p* = 0.002 respectively). There was also a positive correlation with CFU bacterial load (r = 0.506, *p* = 0.005). A bacteria-associated exacerbation (*n* = 13) compared to a non- bacteria-associated exacerbation, had higher NE levels (Log difference 3.873, 95%CI 1.396 to 10.740, *p* = 0.011) (Fig. 2a). Microbiology positive exacerbations were associated with a higher NE compared to microbiology negative exacerbations (Log difference 3.273, 95%CI 1.104 to 9.705, *p* = 0.034) (Fig. 2b). The area under the ROC (95%CI) for identifying a bacteria-associated exacerbation for NE was 0.812 (0.657 to 0.968) with a cut-off of 3034 ng/ml having a sensitivity and specificity of 77%.

### NE exacerbation time course

**Fig. 3:**
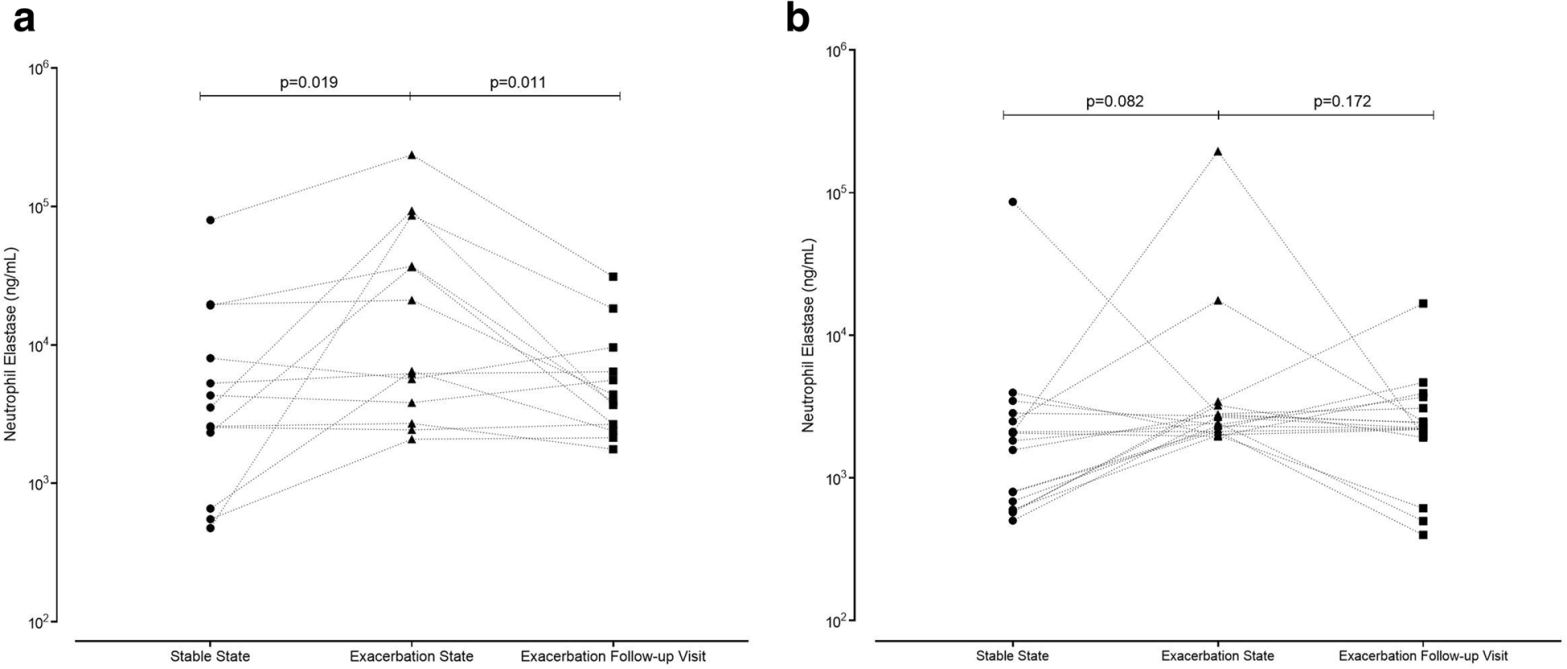
Levels of NE in sputum across an exacerbation time course in subjects with a non-bacterial associated exacerbation (**a**) and those with a bacterial exacerbation (**b**)

NE from stable state, to exacerbation and 2 weeks post treatment time course was studied. A significant difference was seen in NE levels across an exacerbation time course in the subjects with a bacterial exacerbation (*p* = 0.013) (Fig. 3a), but not those with a non-bacterial associated exacerbation (*p* = 0.140) (Fig. 3b). NE levels following an exacerbation returned to baseline for all subjects, irrespective if there was an associated bacterial or non- bacterial exacerbation (Fig. 3a and b).

## Discussion

In this study we have shown that NE levels in patients with COPD are associated with both pulmonary and systemic inflammation, are elevated during an exacerbation, and return to baseline following an exacerbation recovery period. Importantly, this is the first time in patients with a COPD exacerbation, that we could show that NE is a sensitive and specific predictor of a bacteria-associated exacerbation defined as an elevated bacterial load or positive microbiology at the time of an acute event.

COPD is characterised by a neutrophil-dominated airway inflammation, with many subjects having greater than 60% neutrophils detected in their sputum [3]. Neutrophils have many roles within the innate immune system [28]; one such role is defence against invading microorganisms [29]. NE, as well as proteinase 3 and cathepsin G, is a serine protease that is stored in large quantities within neutrophil azurophilic granules [11]. Studies have shown that excesses of serine proteases in sputum have been associated with the pathogenesis of chronic lung diseases [14, 15, 30]. In our study we could show a strong correlation between NE and both systemic and pulmonary neutrophilic inflammation in addition to bacterial load measured by colony forming units. We have also seen that neither neutrophils in the sputum or blood are a viable biomarker for detecting an infection. This is similar to data presented before in a COPD exacerbation study [20]. In our data, NE shows promise for detecting a bacteria-associated infection and importantly a bacteria driven exacerbation; this has not previously been measured before in a COPD exacerbation cohort. NE has been investigated as a biomarker in cystic fibrosis and bronchiectasis patients, looking at disease severity but not specifically as a biomarker for a bacterial exacerbation [16, 17, 31]. The superiority of NE over neutrophils as a biomarker for a bacterial-associated exacerbation could relate to pathogen killing through neutrophil extracellular traps (NETs) [11] as well as being released by neutrophils [8]. NETs produced by neutrophils aid in the killing of pathogens within the airways [32] and have been associated with disease severity in subjects with COPD [33].

*H. influenzae*, a gram-negative bacterium, plays an integral role in both chronic and acute infections in patients with COPD [3], along with other bacteria [34]. NE has been shown to cleave outer membrane proteins of gram-negative bacteria [11], which defines its importance in the host defences against bacteria in COPD subjects as *H. influenzae* is one of the most abundant bacterium in the airways [34]. NE is associated with the killing of pathogens both intracellularly through the direct digestion of phagocytosed microorganisms [35] and through NETs [32]. Although we did not qualify the bacteria during the exacerbation event, our findings that NE is a promising biomarker for a bacteria-associated exacerbation in subjects with COPD supports that this may be irrespective of the underlying pathogen. NE levels have been investigated previously and were shown to be elevated in subjects with COPD compared to healthy controls at stable state [36] and may reflect both chronic infection and inflammation in COPD [37]. In previous analyses, conducted on small numbers of patients, an increase in NE levels at exacerbation have not been observed [38], and previous studies did not investigate links to inflammation of the airways. We could show a significant increase in the active form of NE; this difference is likely related to advances in detection of NE activity. Previous studies in bronchiectasis have linked NE to exacerbations and lung function decline and have suggested that NE could be a potential biomarker for a bacterial infection [16, 31]. Our novel findings support the previous literature and show that NE has a role in another chronic inflammatory pulmonary disease.

There are a few limitations within this study to consider. Firstly, there were no healthy controls included for comparison. However a previous study comparing NE in healthy controls and COPD patients found that NE was highly elevated in patients with COPD [39]. We have added to the existing literature by investigating NE over a clinical exacerbation time course, whilst also studying inflammation in the airways. Secondly, although we were only able to study 30 patients, this is larger than prior studies and is enhanced by the time-course and assessment of repeatability in the active form of NE.

## Conclusion

The data collected indicates that NE levels in patients with COPD are closely related to neutrophilic inflammation and has the potential to be a biomarker indicative of a bacterial associated exacerbation. Whether this could be used to direct antibiotic treatment at the onset of an exacerbation needs further investigation.

## Data Availability

Data not freely available. Study protocol does not allow public sharing of data.

## Abbreviations

CFU: Colony forming units
Cl: Confidence interval
CoE: Coefficient of variation
COPD: Chronic obstructive pulmonary disease
CRQ: Chronic respiratory disease questionnaire
DTT: Dithiothreitol
ECM: Extracellular matrix
FEV_1_: Forced expiratory volume in 1 s
GOLD: Global initiative for chronic obstructive lung disease
ICS: Inhaled corticosteroids
NE: Active neutrophil elastase
PBS: Phosphate-buffered saline
qPCR: quantitative polymerase chain reaction
ROC: Receiver operating characteristic
VAS: Visual analogue scale

## References

[1] Sethi S, Murphy TF (2001). Bacterial infection in chronic obstructive pulmonary disease in 2000: a state-of-the-art review. Clin Microbiol Rev.

[2] Sethi S (2000). BActerial infection and the pathogenesis of copd*. Chest.

[3] Bafadhel M (2015). Airway bacteria measured by quantitative polymerase chain reaction and culture in patients with stable COPD: relationship with neutrophilic airway inflammation, exacerbation frequency, and lung function. Int J Chron Obstruct Pulmon Dis.

[4] Hoenderdos K, Condliffe A (2013). The neutrophil in chronic obstructive pulmonary disease. Too little, too late or too much, too soon?. Am J Respir Cell Mol Biol.

[5] Mantovani A (2011). Neutrophils in the activation and regulation of innate and adaptive immunity. Nat Rev Immunol.

[6] Hamid Q (2002). Methods of sputum processing for cell counts, immunocytochemistry and <em>in situ</em> hybridisation. European Respiratory J.

[7] Singh D, et al. Sputum neutrophils as a biomarker in COPD: findings from the ECLIPSE study. Respir Res. 2010;11(1):77–7. 10.1186/1465-9921-11-77 PMC2904285 20550701

[8] Döring G (1994). The Role of Neutrophil Elastase in Chronic Inflammation. Am J Respir Crit Care Med.

[9] Dau T (2015). Autoprocessing of neutrophil elastase near its active site reduces the efficiency of natural and synthetic elastase inhibitors. Nat Commun.

[10] Pham CTN (2006). Neutrophil serine proteases: specific regulators of inflammation. Nat Rev Immunol.

[11] Korkmaz B (2010). Neutrophil elastase, proteinase 3, and Cathepsin G as therapeutic targets in human diseases. Pharmacol Rev.

[12] Fuentes-Prior P, Salvesen GS (2004). The protein structures that shape caspase activity, specificity, activation and inhibition. Biochem J.

[13] Drescher B, Bai F (2013). Neutrophil in viral infections, friend or foe?. Virus Res.

[14] Watz H (2014). Conduct of a biomarker study in bronchiectasis patients: Correlation of neutrophil elastase activity and inflammatory load in induced sputum. European Respiratory J.

[15] Twigg MS (2015). The role of serine proteases and Antiproteases in the cystic fibrosis lung. Mediat Inflamm.

[16] Chalmers JD (2017). Neutrophil elastase activity is associated with exacerbations and lung function decline in bronchiectasis. Am J Respir Crit Care Med.

[17] Muhlebach MS (2016). Biomarkers for cystic fibrosis drug development. J Cyst Fibros.

[18] Stockley R (2013). Phase II study of a neutrophil elastase inhibitor (AZD9668) in patients with bronchiectasis. Respir Med.

[19] Crocetti L (2019). A patenting perspective on human neutrophil elastase (HNE) inhibitors (2014–2018) and their therapeutic applications. Expert opinion on therapeutic patents.

[20] Bafadhel M (2011). Acute exacerbations of chronic obstructive pulmonary disease: identification of biologic clusters and their biomarkers. Am J Respir Crit Care Med.

[21] Erkan L (2008). Role of bacteria in acute exacerbations of chronic obstructive pulmonary disease. Int J Chron Obstruct Pulmon Dis.

[22] Berkowitz FE (1995). Antibiotic resistance in bacteria. South Med J.

[23] Chauvin A (2008). Outcomes in cardiopulmonary physical therapy: chronic respiratory disease questionnaire (CRQ). Cardiopulmonary Physical Therapy J.

[24] Brightling CE, Monterio W, Green RH, Parker D, Morgan MD, Wardlaw AJ, Pavord ID (2001). Induced sputum and other outcome measures in chronic obstructive pulmonary disease: safety and repeatability. Respir Med.

[25] Anthonisen NR (1987). Antibiotic therapy in exacerbations of chronic obstructive pulmonary disease. Ann Intern Med.

[26] https://www.nice.org.uk, Chronic obstructive pulmonary disease 2016.

[27] Rabe KF (2007). Global strategy for the diagnosis, management, and prevention of chronic obstructive pulmonary disease. Am J Respir Crit Care Med.

[28] Chaplin DD (2010). Overview of the immune response. J Allergy Clin Immunol.

[29] Boyton RJ, Openshaw PJ (2002). Pulmonary defences to acute respiratory infection. Br Med Bull.

[30] Keir HR (2017). Profile of the ProAxsis active neutrophil elastase immunoassay for precision medicine in chronic respiratory disease. Expert Rev Mol Diagn.

[31] Brusselle GG, Braeckel EV (2017). Sputum neutrophil elastase as a biomarker for disease activity in bronchiectasis. Am J Respir Crit Care Med.

[32] Kaplan MJ, Radic M (2012). Neutrophil extracellular traps (NETs): Double-edged swords of innate immunity. J Immunol (Baltimore, Md. : 1950).

[33] Dicker AJ (2018). Neutrophil extracellular traps are associated with disease severity and microbiota diversity in patients with chronic obstructive pulmonary disease. J Allergy Clin Immunol.

[34] Barker BL (2015). Association between pathogens detected using quantitative polymerase chain reaction with airway inflammation in COPD at stable state and exacerbations. Chest.

[35] Belaaouaj A (2002). Neutrophil elastase-mediated killing of bacteria: lessons from targeted mutagenesis. Microbes Infect.

[36] Patel N, et al. Measurement of C-reactive protein, procalcitonin and neutrophil elastase in saliva of COPD patients and healthy controls: correlation to self-reported wellbeing parameters. Respir Res. 2015;16(1):62–2. 10.1186/s12931-015-0219-1 PMC4451749 26018813

[37] Monsó E (1995). Bacterial infection in chronic obstructive pulmonary disease. A study of stable and exacerbated outpatients using the protected specimen brush. Am J Respir Crit Care Med.

[38] Chillappagari S, et al. Altered protease and antiprotease balance during a COPD exacerbation contributes to mucus obstruction. Respir Res. 2015;16(1):85–5. 10.1186/s12931-015-0247-x PMC4501272 26169056

[39] Baines KJ, Simpson JL, Gibson PG (2011). Innate immune responses are increased in chronic obstructive pulmonary disease. PLoS One.

